# Transcriptome bioinformatic analysis identifies potential therapeutic mechanism of pentylenetetrazole in down syndrome

**DOI:** 10.1186/1756-0381-3-7

**Published:** 2010-10-28

**Authors:** Abhay Sharma

**Affiliations:** 1 Institute of Genomics and Integrative Biology, Council of Scientific and Industrial Research Delhi University Campus, Mall Road, Delhi 110007, India

## Abstract

**Background:**

Pentylenetetrazole (PTZ) has recently been found to ameliorate cognitive impairment in rodent models of Down syndrome (DS). The mechanism underlying PTZ's therapeutic effect in DS is however not clear. Microarray profiling has previously reported differential expression, both up- and down-regulation, of genes in DS. Given this, transcriptomic data related to PTZ treatment, if available, could be used to understand the drug's therapeutic mechanism in DS. No such mammalian data however exists. Nevertheless, a *Drosophila *model inspired by PTZ induced kindling plasticity in rodents has recently been described. Microarray profiling has shown PTZ's downregulatory effect on gene expression in the fly heads.

**Methods:**

In a comparative transcriptomics approach, I have analyzed the available microarray data in order to identify potential therapeutic mechanism of PTZ in DS. In the analysis, summary data of up- and down-regulated genes reported in human DS studies and of down-regulated genes reported in the *Drosophila *model has been used.

**Results:**

I find that transcriptomic correlate of chronic PTZ in *Drosophila *counteracts that of DS. Genes downregulated by PTZ significantly over-represent genes upregulated in DS and under-represent genes downregulated in DS. Further, the genes which are common in the downregulated and upregulated DS set show enrichment for MAP kinase pathway.

**Conclusion:**

My analysis suggests that downregulation of MAP kinase pathway may mediate therapeutic effect of PTZ in DS. Existing evidence implicating MAP kinase pathway in DS supports this observation.

## Background

Chronic treatment with nonconvulsive dosage of PTZ has recently been found to ameliorate cognitive impairment in rodent models of DS [1-4]. The mechanism underlying PTZ's potential therapeutic effect in DS is however unclear. Genome scale expression analysis offers a promising approach to identify genes and pathways relevant in pathophysiological and therapeutic mechanisms in complex CNS disorders [5]. Microarray gene expression profiling has previously been reported in the analysis of control versus DS astrocyte cell line and cerebrum or apical frontal pole [6], prefrontal cortex [7], and neural progenitor cells [8]. However, transcriptomic analysis of effect of PTZ in mammalian system has not been undertaken yet. This precludes understanding drug's potential mechanism using functional genomic data.

A *Drosophila *model inspired by rodent models of chronic PTZ induced kindling plasticity has recently been developed [9]. In this model, PTZ causes a decreased speed in startle-induced climbing in flies. Antiepileptic drugs, used in treating epilepsy and other neurological and psychiatric disorders, suppress development of this behavioral deficit. Microarray profiling has shown that PTZ exerts a downregulatory effect on gene expression in fly heads. This effect has been found to mimic transcriptome and proteome scale changes reported previously in human epilepsy patients and mammalian models of epileptogenesis. Given the above, the fly model provides a systems level framework for understanding potential disease and drug mechanisms [9]. In a comparative transcriptomics approach, I examine here if mining of the available fly [9] and human [6-8] microarray data could uncover potential mechanism of PTZ action in DS.

## Methods

Chronic PTZ regulated *Drosophila *genes, all downregulated, listed in Additional File three of the previous report [9], were used in the analysis. Literature on relevant microarray profiling in DS was searched in PubMed http://www.ncbi.nlm.nih.gov/pubmed. For DS versus control microarrays, differentially expressed genes listed in Supplementary Tables five a, five b, six a and six b of Mao et al.'s paper [6], Supplementary Table three of Lockstone et al.'s paper [7], and Supplementary Data (≥1.5 fold) of Esposito et al.'s paper [8] was used. These human studies involved microarray expression analysis of developing brain, adult brain, and neural progenitors, in that order. Overlap between gene sets and pathway enrichment was examined using hypergeometric distribution probability in excel. The overlap was considered statistically significant if the nominal *p *value was found to be less than 0.05. Human homologs (gene symbols) of *Drosophila *genes were retrieved using Homologene option in FLIGHT http://www.flight.licr.org/search/batch_homology.jsp. Gene IDs described in human studies were converted to gene symbols using DAVID http://david.abcc.ncifcrf.gov/summary.jsp, NCBI http://www.ncbi.nlm.nih.gov/unigene/ and SOURCE http://smd.stanford.edu/cgi-bin/source/sourceBatchSearch. Genes were depicted in the KEGG pathway for *Homo sapiens *http://www.genome.jp/kegg/tool/color_pathway.html.

## Results

**Figure 1:**
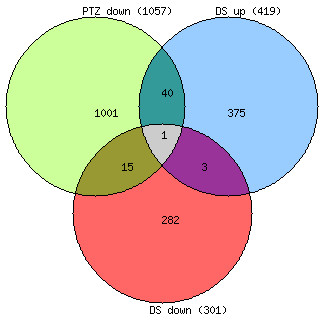
**Venn diagram showing overlaps among PTZ and DS genes**. Of the 716 total up- and down-regulated genes in DS, 56 are common to the PTZ downregulated set. Of the 419 upregulated DS genes, 41 are common to the PTZ set. Of the 301 downregulated DS genes, 16 are common to the PTZ set. Note significant enrichment in PTZ and DS total versus PTZ and DS upregulated (*p *= 0.011) and depletion in PTZ and DS total versus PTZ and DS downregulated (*p *= 0.008).

**Figure 2:**
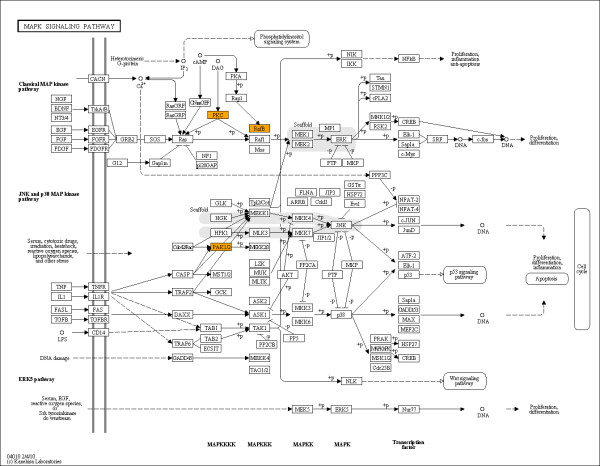
**MAP kinase pathway showing counteracting commonality genes**. Of the 419 DS upregulated genes, 9 mapped on to the pathway. Of the 41 counteracting commonality genes, i.e., genes common between PTZ downregulated and DS upregulated sets, 3 figured in the pathway map (BRAF, PAK1 and PRKCA; represented by the three orange color boxes). Note significant enrichment of MAP kinase pathway in counteracting commonality genes (*p *= 0.041).

I first examined if PTZ regulated genes in *Drosophila *[9] counteract differentially expressed genes in DS [6-8]. The three diverse DS studies reported differentially expressed genes with insignificant overlap. Genes in DS studies were thus pooled together for matching with human homologs of PTZ regulated genes (for gene lists, see additional file 1). Strikingly, a significantly higher overlap was found between genes downregulated by PTZ and genes upregulated in DS, and a significantly lower overlap between downregulated genes in PTZ and DS sets (Figure 1). Enrichment for MAP kinase pathway in DS upregulated genes has previously been reported [8]. In contrast, downregulated genes in *Drosophila*, the only regulated genes in the PTZ model, have been found to enrich MAP kinase pathway [9]. Thus, I next predicted that significant overlap between PTZ downregulated and DS upregulated genes may result from counteracting effect on MAP kinase signaling. Remarkably, the counteracting commonality genes between PTZ downregulated and DS upregulated sets were found to enrich the MAP kinase pathway (Figure 2). Together, my transcriptomic analysis provided evidence for the involvement of MAP kinase pathway in the mechanism of action of PTZ in DS.

As the above enrichment analysis was biased for MAP kinase pathway due to the prior hypothesis regarding its possible involvement, I next examined if evidence for this pathway is further supported in an unbiased analysis. As in the above MAP kinase analysis, counteracting commonality genes between PTZ downregulated and DS upregulated sets were compared against total DS upregulated genes. Six additional pathways were found enriched in this analysis - Metabolic pathways (6 vs. 36 genes; *p *= 0.0008), Focal adhesion (3 vs. 9 genes; *p *= 0.041), ErbB signaling pathway 3 vs. 7 genes; (*p *= 0.02), Arginine and proline metabolism (3 vs. 7 genes; *p *= 0.02), Natural killer cell mediated cytotoxicity (3 vs. 5 genes; *p *= 0.007), and Vascular smooth muscle contraction (3 vs. 7 genes; *p *= 0.02). Interestingly, three of these pathways are directly connected to MAP kinase pathway in the KEGG database. As such, this finding supported the centrality of MAP kinase pathway in therapeutic action of PTZ in DS.

## Discussion

I have used two diverse set of summary data of transcriptomic profiling - related to three separate human DS studies [6-8] and to the description of *Drosophila *PTZ model [9] - to examine if potential mechanism(s) of drug action can be identified through bioinformatics. The human studies reported differentially expressed genes in DS brain in different context, namely, developing brain [6], adult brain [7], and neural progenitors [8]. Not surprisingly, as mentioned above, no significant match was observed between genes reported in these disparate studies. In contrast to human studies, the *Drosophila *data pertained to expression profiling of whole CNS from PTZ treated animals. Despite potential confounding effects - arising out of tissue, species and experimental diversity - in the reported data, I analyzed the data under the assumption that genes in diverse human studies when combined together would capture a broader spectrum of expression changes in DS and hence be appropriate enough for uncovering potential PTZ mechanism. Given this, it is striking that downregulation of MAP kinase signaling pathway was identified as a potential therapeutic mechanism by which PTZ may act in DS. This finding, notably, is supported by existing evidence from diverse studies. For example, protein analysis of fetal brain cortex has previously identified dysregulation of MAP kinase pathway related components in DS [10]. Also, comparative genomics analysis has predicted perturbation in MAP kinase pathway in DS [11]. Further, biochemical analysis has suggested a role of activated MAP kinase signaling in brain pathogenesis in mouse DS model [12]. Besides, bioinformatic analysis of genes located in the candidate DS region in chromosome 21 has implicated MAP kinase pathway in the disease [13]. Biochemical, genomic and computational evidence thus exist to support the plausibility of MAP kinase signaling as PTZ's therapeutic target in DS.

## Conclusion

Bioinformatic analysis of human and *Drosophila *transcriptome suggests that downregulation of MAP kinase pathway may underlie therapeutic effect of pentylenetetrazole in ameliorating cognitive impairment in DS. This warrants experimental validation in rodent models of DS.

## Competing interests

The author declares that he has no competing interests.

## Supplementary Material

Additional file 1 **Differentially expressed genes reported in *Drosophila *model and DS studies**. List of up- and/or down-regulated genes reported by Mohammad *et al.*, Esposito *et al.*, Lockstone *et al. *and Mao *et al.* Click here for file
